# Light activation of the dopaminergic system occurs after eye-opening in the mouse retina

**DOI:** 10.3389/fopht.2023.1184627

**Published:** 2023-05-09

**Authors:** Vrinda Jain, Phillip J. M. Liang, Sushmitha Raja, Meena Mikhael, Morven A. Cameron

**Affiliations:** ^1^ School of Medicine, Western Sydney University, Sydney, NSW, Australia; ^2^ Mass Spectrometry Facility, Western Sydney University, Sydney, NSW, Australia

**Keywords:** retina, dopamine, development, c-fos, melanopsin

## Abstract

The neuromodulator dopamine plays a significant role in light adaptation, eye growth, and modulation of neuronal circuitry in the retina. Dopaminergic amacrine cells in the adult retina release dopamine in response to light stimulation, however, the light-induced activity of these cells in during postnatal development is not known. We assessed the activity of dopaminergic amacrine cells in the retina response to a light pulse in C57BL/6 wild-type animals across various postnatal ages. Expression of tyrosine hydroxylase (TH) in dopaminergic amacrine cells was apparent from postnatal day 3 (P3) and restricted to the dorso-temporal region; by P8 TH+ cells were uniformly distributed across the retina. TH cell density increased until P8 and then markedly decreased by P10 to then remain at this density into adulthood. Light-induced c-fos expression was observed in all light-pulsed retinae, however, no c-fos was ever found to be co-localised with TH prior to P12. At P14, one day after eye opening, 100% of TH cells co-localised with c-fos and this was maintained for all older ages analysed. Dopamine and its primary metabolite DOPAC were measured in the vitreous of animals P8-P30. Both analytes were found in the vitreous at all ages, however, a significant difference in dopamine concentration between dark and light-pulsed animals was only observed at P30. DOPAC concentration was found to be significantly light-induced from P16, and the amplitude of this difference increased over time. Our data suggests that dopaminergic cell activation and light-induced dopamine release in the retina is primarily driven by classical photoreceptors after eye-opening.

## Introduction

The neuromodulator dopamine plays a significant role in the mammalian retina driving light adaptation by modulating retinal circuitry (1–3). Dopamine has also been shown to be important during retinal development and eye growth (4) and is released in response to retinal waves (5). Released by a subset of dopaminergic amacrine cells (A18) that reside in the inner nuclear layer, dopamine diffuses throughout the layers of the retina and acts on both D1- and D2- type dopamine receptors that are expressed on every class of retinal cell (reviewed in (6)).

We have recently shown that light-induced dopamine release in the adult mouse retina relies primarily on rod phototransduction (7). However, dopaminergic cells are present in the retina in early development (5, 8) before the maturation of rod photoreceptors or the synaptic circuitry that might convey light signals to these cells (9, 10). In contrast, intrinsically photoresponsive retinal ganglion cell (ipRGC) photoreceptors have been shown to influence retinal development before eye-opening and are functional even before birth (5, 11, 12). Intriguingly, Munteanu et al, showed that dark rearing reduced the overall number of dopaminergic cells and dopamine content of the retina at postnatal day 14 (P14) and into adulthood in mice. Furthermore, they show that it is rod phototransduction specifically that is required to produce normal development in response to standard cyclical lighting conditions of the animals (12). Together, these data suggest that light inputs to dopaminergic cells are predominately rod-driven, both in development and adulthood. However, the influence of ipRGCs on dopaminergic cell activation prior to the maturation of other photoreceptor circuitry, remains to seen.

Here we assess the normal development of dopaminergic amacrine cells, both pre- and post-eye-opening, and their activation and release of dopamine in response to light. We show that while dopaminergic cells begin to express tyrosine hydroxylase (TH; rate-limiting enzyme in dopamine production) by P3, activation of these cells (assessed by c-fos expression) or dopamine release in response to light does not occur until after eye-opening. However, c-fos expression in non-TH expressing cells in response to light is widespread in the retina and is driven exclusively by melanopsin phototransduction before P12.

## Methods

### Animals

Animal care was in accordance with the Australian Code for the Care and Use of Animals for Scientific Purposes. Protocols were approved and monitored by the Western Sydney University Animal Care and Ethics Committee, project numbers: A12402 and A14720. Wild-type C57BL/6J (ARC, Canning Vale, Australia) and *Opn4^cre/cre^* (mixed C57BL/6 and 129sv; (13)) mice were bred on site. In *Opn4^cre/cre^* animals, the cre locus replaces that of melanopsin making these melanopsin-deficient animals which will hereafter be referred to as *Opn4^-/-^*. Offspring (both male and female) P3 - P30 days were used. Unless otherwise stated, animals were maintained under a 12hr light:12hr dark cycle at 300 lux illumination during the light phase. Animals were checked once per day and birthday was recorded; however, exact birth time was not recorded and so may have varied between litters. We endeavoured to include animals of the same litter in each experimental time-point, but this was not always possible. In our hands, animals up to, and including, P12 still had closed eyes; all animals > P14 had open eyes.

### Light-pulsing and tissue removal

To assess light-activation and dopamine release from TH cells, prior to tissue removal, all animals were dark-adapted from dusk the preceding day until subjective midday (CT6 ± 1.5 hrs). Following dark-adaptation free-moving mice at various postnatal ages (P3, P8, P10, P12, P14, P16, P19, P30) were light pulsed with broadband white light (16.4 log photons cm^-2^ s^-1^; ~26,000 lux at cage floor; see spectrum in Supp Data 1) for either 90 minutes (c-fos experiments) or 1 hr (LC-MS) in their home cages with their parents. Tissue was removed immediately after this light pulse under the same lighting conditions. Littermate dark control animals were sacrificed at the same circadian time (CT6 ± 1.5 hrs) and tissue removed under infrared conditions (> 900 nm; -11.9 log scot cd^-1^ m^-2^ at animal).

Eyes for immunohistochemistry were enucleated with curved scissors, the cornea and lens were removed, placed in 1.5mL tubes of 4% paraformaldehyde for 1 hour, and then transferred into 0.01M PBS. The orientation of each retina was marked prior to staining using the choroidal fissure as a guide (14). For dopamine and DOPAC quantification, vitreous was removed by piercing eyes through the ora serrata into the vitreal chamber with a 27G needle and squeezing the resulting fluid onto a plastic petri dish. 2 μl was then pipetted into 48μL of 0.5 mM ascorbic acid (Sigma) containing internal standards 2-(3,4-dihydroxyphenyl)ethyl-1,1,2,2- d4-amine hydrochloride 98% (dopamine-d4; CDN isotopes; D-1540) and 3,4-didroxyphenylacetic acid ring-d3, 2,2-d2, 98% (DOPAC-d5; Cambridge Isotope Laboratories; DLM-2499) at 10 ng/ml for each.

### Immunohistochemistry

Both retinal wholemounts and slices were prepared. Wholemounts were dissected from the eyecup and stained free-floating in 0.01M PBS. Eyes used for slices were placed in 30% sucrose 0.01M PBS solution overnight for cryoprotection and then submerged in OCT medium and frozen with liquid nitrogen. The frozen eyes were then brought to -20°C and 30 μm sections cut and adhered to gelatine coated slides. The slides were then incubated at 37°C for 2 hours before being stored at 4°C for immunohistochemistry.

All incubation steps took place at 4°C and were performed in 0.3% Triton-X-100 in 0.01M PBS. Samples were blocked with 5% donkey serum for 2 hours, then incubated with primary antibodies: sheep anti-TH (1:500; Merck Millipore; AB1542), rabbit anti-c-fos (1:500; Cell Signaling; 9F6), and chicken anti-Opn4 (1:5000; a kind gift from Dr. MTH Do, Harvard Medical School (15)), in 1% donkey serum for 3 days. Samples were then washed with 0.01M PBS (1 initial followed by 2 x 5-minute washes, and 1 final 1-hour wash), and then incubated with secondary antibodies all raised in donkey: anti-sheep Alexa 488 (713-545-003), anti-rabbit Cy3 (711-165-152) and anti-chicken Alexa 647 (703-605-155; Jackson ImmunoResearch), in 2% donkey serum for 3 hrs. Retinae were washed using the same method as above. Wholemount retinae were mounted onto gelatine coated slides and both slices and wholemount were coverslipped in Prolong-X Gold mounting media (Thermo Fisher Scientific; P10144), and left to cure overnight and then stored at 4°C until analysis.

### Microscopy

TH cell and c-fos density was quantified using a Zeiss AxioImager M2 microscope with MBF Biosciences StereoInvestigator. A contour was drawn around the retina with the 5x objective, and the counting frame size used was 500 x 500 μm. Total number of TH positive cells, c-fos, and TH/c-fos colocalised cells were counted through the entire thickness of the tissue using the 20x objective. These values were then divided by the area of the contoured retina to obtain a density value of cells per mm^2^. Representative retinal images were taken using a Zeiss Airyscan 800; wholemount retinal images were shallow stacks (~20 μm) taken through the inner nuclear layer and stratum 1 of the inner plexiform layer to show TH cell bodies and processes.

### LC-MS methods

Mass spectrometric detection was performed on the Sciex Triple Quad™ 7500 mass spectrometer, fitted with an electrospray ionisation source (OptiFlow Pro Ion Source). MRM scan parameters for dopamine (parent 154.22; fragment 91.01) were: entrance potential (EP) 10V; collision energy (CE) 33V; collision cell exit potential (CXP) 12V. Dopamine-d4 (parent 158.22; fragment 141.03, EP 10V, CE 34V, CXP 11V), DOPAC (parent 123.04, daughter 40.973, EP -10V, CE -26V, CXP -20V), DOPAC-d5 (parent 128.1, fragment 100.0, EP -10V, CE -16V, CXP -7V). Parameters were as follows: curtain gas (CUR), 40 psi; ion source gas 1 (Gas1), 70 psi; ion source gas 2 (Gas2), 70 psi; CAD gas, 9; temperature (TEM), 450°C.

Liquid chromatography was performed using a Waters Acquity I-Class+ UPLC, working at a flow rate of 0.3mL/min with a Waters Acquity UPLC HSS C18 column of 1.7μm particle size, 2.1 × 100mm. The mobile phases were 0.1% (v/v) formic acid in Milli-Q water (A) and LC-MS grade methanol (B) at a gradient of 0-0.5min: 5% B; 0.5-3min: 5-100% B; 3-6min: 100% B; 6-7min: 5% B. Column and sample manager temperatures were kept at 40°C and 4°C respectively. Injection volume was 10μL in full loop mode (overfill factor of 3) from sample solutions contained in Total Recovery (Waters) glass vials. Dopamine (retention time - RT = 1.00 min) and dopamine-d4 (RT = 1.00 min) were analysed in positive ion mode whereas DOPAC (RT = 2.30 min) and DOPAC-d5 (RT = 2.30 min) were analysed in negative ion mode.

## Results

### Cell density and distribution

**Figure 1:**
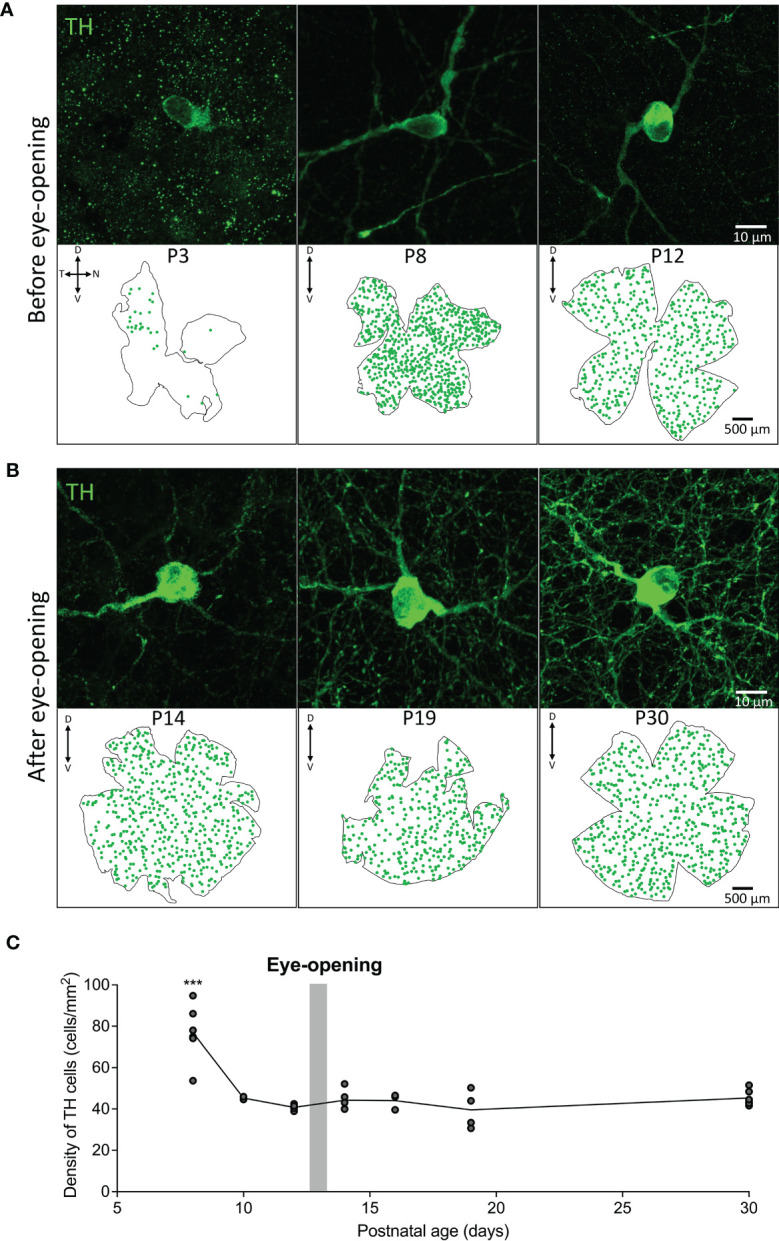
Density and distribution of dopaminergic cells before and after eye-opening. **(A)**, Representative images from wholemount retina before eye-opening show TH (green; dopaminergic amacrine cells) staining from postnatal day 3 (P3) in the dorsal temporal retina. TH cell distribution appears across the entire retina at P8 and P12. **(B)**, TH cells in the wholemount retina after eye-opening appear evenly distributed throughout the retina. **(C)**, Quantification of cell densities from P8 shows a significant decline in TH cell density after P8 before stabilising to adult TH cell density levels (one-way ANOVA with Tukey post-test ***P < 0.001). P8 n=5; P10 n=2; P12 n=4; P14 n=4; P16 n=3; P19 n=4; P30 n=5. Grey bar – age of eye-opening (P13). Dorsal, ventral, nasal and temporal marked as D, V, N and T respectively.

The density and distribution of tyrosine hydroxylase positive dopaminergic amacrine cells (TH cells) was assessed across development before and after eye-opening in animals raised under normal lighting conditions. TH positive cells were first observed from P3 and were found to be primarily in the dorso-temporal region of the retina (**Figure 1A**). The number of TH cells increased from P3 to a peak at P8 which can be visualized in the retinal maps shown in **Figure 1A**. The distribution of TH cells across the retina became uniform by P8, with an average density of 76.9 ± 5.6 cells/mm^2^ with no obvious concentration gradients or variations in density. Consequently, average densities are plotted after P8 in **Figure 1C**. Interestingly, between P8 and P10, prior to rod photoreceptor circuitry maturation, there was a significant decrease in this cell density to 45.3 ± 0.7 cells/mm^2^ (one-way ANOVA with Tukey post-test ***P < 0.001; **Figure 1C**). While a decrease in density could be due to growth of the retinal tissue, there was also a decrease in the total number of TH cells from 740 ± 28 cells at P8 to 563.5 ± 6.5 cells at P10 (*P<0.05; unpaired Student’s t-test, n=2 for each). The cell density then did not change into adulthood remaining around 40-45 cells/mm^2^ (one-way ANOVA with Tukey post-test P > 0.9 for all comparisons P10-P30; **Figure 1C**).

### C-fos activation of dopaminergic cells by light is tightly linked to eye-opening

**Figure 2:**
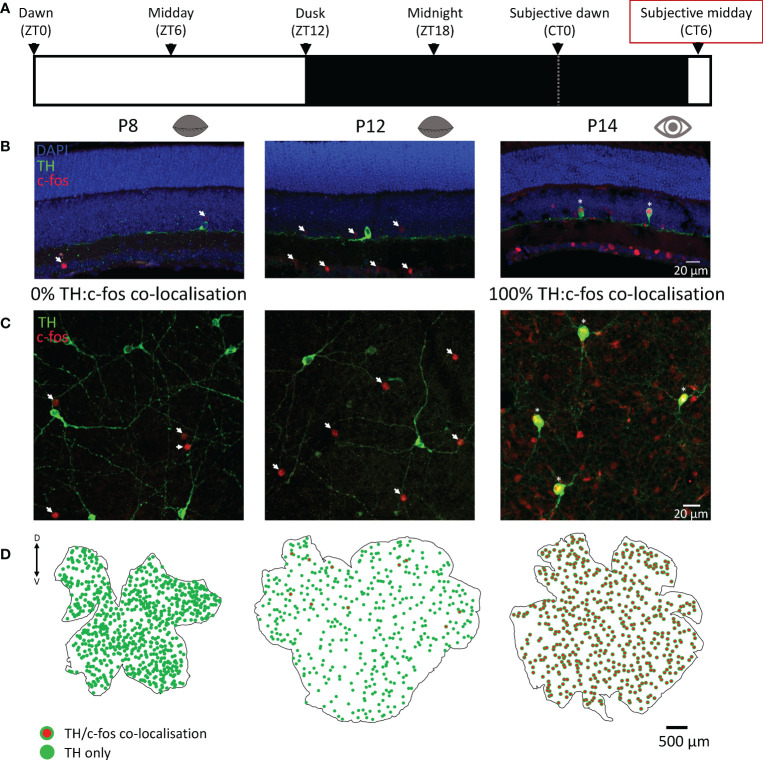
C-fos activation by light in dopaminergic cells is tightly linked to eye-opening. **(A)**, Schematic representation of the light environment of the animals prior to light-pulsing. Animals were dark-adapted from dusk the preceding day and light-pulsed for 90 min at subjective midday (red box; CT6 ± 1.5 hrs), then ocular tissues were immediately removed. **(B, C)**, Representative images of sliced and wholemount light-pulsed retinae show TH (green; dopaminergic) cell staining with 0% colocalisation with c-fos (red) at postnatal day 8 (P8), before eye opening, and 100% colocalization with c-fos (red) at P14, after eye opening. **(D)**, Retinal maps showing TH (green) and TH and c-fos colocalization (red with green border) with no colocalization at P8 (left), a small subset of colocalised cells in the dorsal retina at P12 (middle), and complete colocalization at P14 (right), immediately after eye opening. Arrows: c-fos only; asterisks: c-fos/TH colocalization; dorsal and ventral marked as D and V respectively.

Dopaminergic amacrine cells are known to depolarise and release dopamine in response to light in the adult retina. Here we used c-fos, a marker of neuronal depolarization (16), to determine if light influences the activity of these cells in response to a 90 min light pulse. Before P12, no c-fos was ever found co-localised with TH cells in response to light-activation. However, widespread c-fos activation was observed in non-TH cells, but only in light-pulsed retinae (**Figure 2**); no c-fos was observed in any dark control retinae (**Supplementary Figure 1**). C-fos expression in TH cells *was* observed at P12 despite the animal having not yet opened their eyes but was limited to a small subset of cells in the dorsal retina (**Figure 2C**). Interestingly, two different litters of animals light-pulsed at P12 were compared (n=2 for each), in the first litter only 1 TH cell was found to be co-localised with c-fos in each animal. However, in the second litter (possibly a few hours older) 10 and 12 co-localised TH/c-fos cells were found in each animal respectively (**Figure 2C**). This suggests that dopaminergic cell activation by light starts immediately prior to eye-opening and is tightly linked to developmental age. All c-fos+ TH cells in both P12 litters were found in the dorsal retina. By P14 (after eye-opening) 100% of TH cells expressed c-fos in response to light and this 100% co-localisation was observed in all light-pulsed retinae after this age (P16 - P120; data not shown).

### Light-induced dopamine and DOPAC release occurs after eye-opening

**Figure 3:**
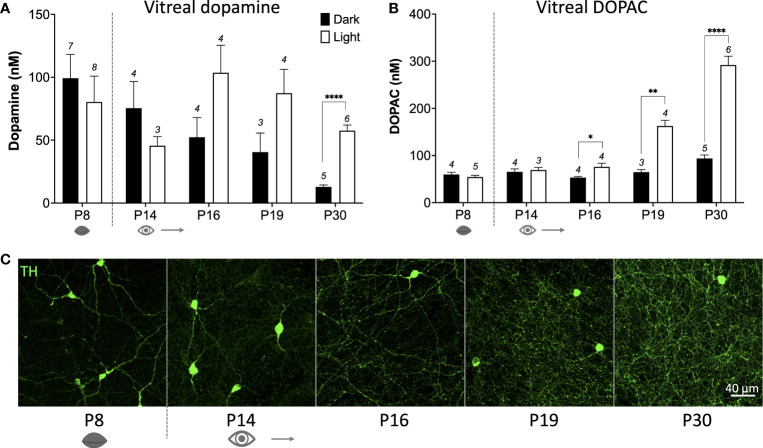
Light-induced dopamine and DOPAC release occurs after eye-opening. **(A)**, Dark-adapted vitreal dopamine concentration decreases with increasing age (P < 0.05; one-way ANOVA). Vitreal dopamine levels are significantly higher in response to a 1hr light pulse compared to dark at postnatal day 30 (P30) (****P < 0.01; unpaired Student’s t-test; n number depicted above columns), but not at any other ages. **(B)**, Vitreal DOPAC (dopamine metabolite) concentration is significantly higher in the light compared to the dark from P16 (P16 *P < 0.05; P19 **P < 0.01; P30 ****P < 0.01; unpaired Student’s t-test). **(C)**, TH (green) cell dendritic axonal plexus complexity increases with age, particularly from P16, with ring structures visualised at P19. Grey dotted line denotes eye-opening.

How does c-fos activation of TH cells correlate with actual release of dopamine from these cells? To answer this question, we measured vitreal dopamine and DOPAC (a dopamine metabolite) which has been shown to be a reliable index of retinal dopamine release (17). We measured from various postnatal ages between P8 to P30 (prior to P8 vitreal volumes were too small to accurately quantify). Vitreous was removed after ~18hrs dark-adaptation at subjective midday under either dark conditions, or after a 1hr light pulse (see schematic **Figure 2A**). In the dark, basal dopamine levels reduce from P8 to P30 (**Figure 3A**; *P < 0.05; one-way ANOVA), whereas DOPAC levels remained at a constant low level in the dark at all ages measured (**Figure 3B**). This suggests that basal dopamine levels may play an important role in development. However, no significant difference was observed in vitreal dopamine concentration between light-pulsed and dark conditions until P30 (****P < 0.0001; unpaired Student’s t-test; **Figure 3A**) although there is a trend for higher dopamine levels in the light at P16 and P19. Vitreal DOPAC concentration is significantly higher in light-pulsed than the dark-adapted eyes after P16 and the magnitude of this difference increases with age (P16 *P < 0.05; P19 **P < 0.01; P30 ****P < 0.0001; unpaired Student’s t-test; **Figure 3B**). We see a considerable increase in the complexity of the TH cell dendritic/axonal processes with age (**Figures 1A, B**, **3C**), particularly from P16 to P30. By P19, TH cell processes span the entire area of the retina, encircling neighbouring neurons with an intricate net structure. This increase in complexity appears to correlate with the ability of the dopaminergic cells to release dopamine in response to light. These data also indicate that vitreal DOPAC is perhaps a more sensitive indicator of light-induced dopamine release than vitreal dopamine which is more variable, in both light and dark conditions, throughout development in comparison to the adult retina.

### Melanopsin drives retinal c-fos before eye-opening

**Figure 4:**
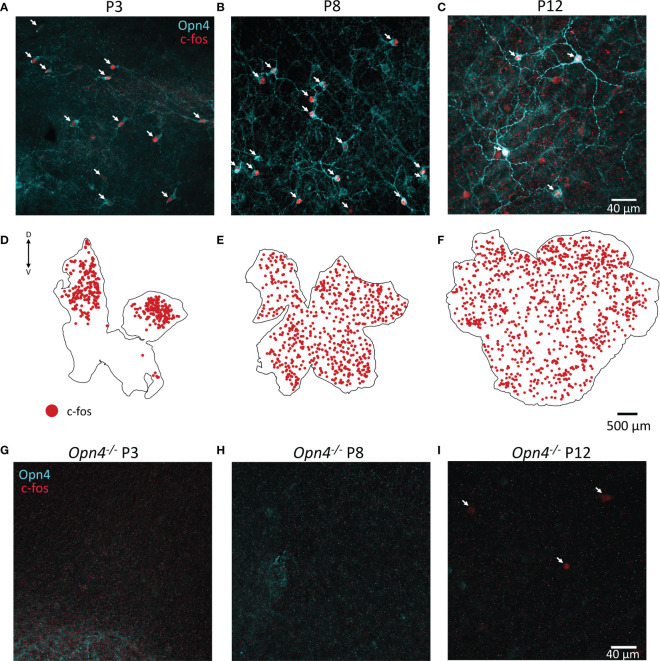
Retinal c-fos is driven by melanopsin before eye opening. **(A, B)**, Representative images of wholemount retinae P3 and P8 show c-fos (red) and melanopsin (Opn4, cyan) colocalization in the majority of cells after a 90 min light pulse. **(C)**, Colocalization of Opn4 and c-fos is also seen at P12 but more non-colocalised cells are observed. **(D)**, C-fos expression is restricted to the dorsal retina at P3, and throughout the retina at P8 and P12 **(E, F)**. Dorsal and ventral marked as D and V respectively. **(G–I)**, No c-fos was observed in Opn4-deficient mice at P3 or P8; some c-fos could be observed at P12 (arrows).

As c-fos activation is widespread in the retina prior to eye-opening, albeit specifically not in TH cells, we co-stained retinae at P3, P8 and P12 for melanopsin. We found that a substantial number (but not all) of the c-fos cells co-localised with melanopsin (arrows **Figures 4A–C**). Since antibodies against melanopsin are known to only reveal a subset of ipRGCs, we repeated the experiment in animals lacking melanopsin (*Opn4^-/-^*). We did not observe any c-fos in light-pulsed retinae in *Opn4^-/-^* animals at P3 or P8, whereas some c-fos+ cells were evident at P12 (**Figure 4G–I**). It is likely that this c-fos at P12 reflects activation from recently mature rod or cone photoreceptors. Therefore, all c-fos observed in wild-type retinae at P3 and P8 (**Figures 4A, B** and retinal maps in **Figures 4D, E**) is driven by melanopsin phototransduction. Surprisingly, c-fos activation at P3 was restricted to the dorsal retina (**Figures 4D**), whereas melanopsin staining could be observed all over including the ventral retina (data not shown); by P8, c-fos was homogenously expressed across the entire retinal area (**Figures 4E, F**).

## Discussion

Our observation that dopaminergic cells begin to express TH at ~P3 is consistent with previous reports (5, 18), although it is thought that these cells are differentiated much earlier, before embryonic day 20 (19). Whilst populations of these cells in development have been studied in various mammalian models including rabbit, cat, hamster, gerbil, and mouse (8, 20–22), the significant peak in TH cell density we see at P8, and subsequent drop at P10 prior to eye opening, has not been explicitly reported. This reduction in cell numbers may be due to either cell phenotype changes, or *via* developmental neuronal loss that generally occurs in the mouse retina in the first 2 weeks (23, 24). Certainly, mice lacking the pro-apoptotic *Bax* gene exhibit 5-fold more dopaminergic cells than littermate controls (25) suggesting the reduction between P8 and P10 likely reflects cell apoptosis. Whether this process is modulated by light or not is unknown, but given that dark reared animals show a reduction in dopaminergic cell number, or at least TH expressing cells (12), light may potentially cause a reduction in TH cell apoptosis. Future studies will examine this density change from P8 to P10 under altered light rearing conditions.

The emergence of TH expressing dopaminergic cells in the dorso-temporal region has been previously seen in gerbils and hamsters (20) and appears to correlate with the predominately dorsal confinement of c-fos activation (driven by melanopsin) at P3 (**Figure 4D**). While we find no evidence of direct activation of TH cells by ipRGCs, it is certainly possibly that maturation of dopaminergic cells relies on indirect cues from the activity of ipRGCs. Neither melanopsin, nor ipRGCs themselves, appear to be needed for normal adult development of the dopaminergic system (12) but the pattern of localisation of TH cells has not been described prior to eye-opening in animals lacking melanopsin/ipRGCs. It is possible that the dorso-temporal localisation of TH cells at P3 is dependent on ipRGC activity in early development. Certainly, the correct lamination of cones appears to be directed by melanopsin phototransduction prior to eye opening, but light inputs from rods and cones are able to correct these defects at eye-opening such that the adult retina of *Opn4^-/-^* animals exhibits normal cone lamination (26). Future work will assess the influence of melanopsin phototransduction on the development and localisation of dopaminergic cells at early developmental stages.

The co-localisation of c-fos in TH cells at eye-opening agrees with our previous work that implicates classical photoreceptors, and in particular rods, in driving dopaminergic cell activation by light (7, 27). While the exact neuronal response that drives c-fos activation in retinal cells is not entirely known, it is usually observed in response to strong neuronal depolarisation that allows substantial calcium-influx (16). The ubiquitous light-induced c-fos expression in TH cells following classical photoreceptor circuitry maturation (9, 10) argues that TH cells are robustly activated by rod and/or cone input. If a light-driven synaptic input from ipRGCs to TH cells is present before eye-opening it must not be sufficient to raise calcium levels to the appropriate levels to stimulate c-fos expression. Indeed, while a light-driven ipRGC input to TH cells has been described in adult animals lacking rods and cones (28) we have never observed light-induced nuclear c-fos activation in TH cells in these animals (27). Importantly, light-induced dopamine release has also never been observed in rodless coneless animals (7, 27) suggesting that c-fos activation and dopamine release are correlated. The light intensity delivered to the animals approximates that of a cloudy day at noon. While this is likely somewhat brighter than young pups residing in a nest would experience naturally, we aimed to use an intensity that was sufficient to determine if ipRGCs convey light information to TH cells. Even at these bright intensities, we show here that no TH cells are activated by light before rod and cone maturation.

Vitreal DOPAC shows a small, but significant, light-induced increase at P16, but a light-induced increase in vitreal dopamine is not significant until P30. While there is a trend for increased dopamine under light conditions at P16 and P19, the large variability in dopamine concentration between animals may mask the influence of light on dopamine release. It is known that retinal waves drive dopamine release in the retina before eye-opening (5), and thus, depending on when the animal is sacrificed it may be that a retinal wave had either just occurred, or was just about to occur. This would add significant variability to vitreal dopamine measurements taken before eye-opening but does not explain the variability observed at P14-P19. The answer perhaps lies in the production of DOPAC, a metabolite of dopamine produced intracellularly. While DOPAC is present in the eye prior to eye opening, its concentration is very low and it is not produced in response to light until P16, and even then, at low levels (**Figure 3B**). It is possible that, in contrast to adult retina, dopamine release from TH cells in development is not immediately taken back up *via* the dopamine transporter (DAT). This would lead to a scenario where an overall high dopamine concentration in the retina is maintained at a steady state by frequent retinal waves. It has been shown that the activity of DAT may be driven by violet light *via* neuropsin (Opn5) in development (29), so the lack of violet wavelengths in our standard LED lighting may confound these results. However, it is surprising that widespread activation of ipRGCs with light (**Figures 4A, B**) does not increase dopamine levels when we consider that retinal waves have been shown to increase in duration by up to 50% following light stimulus (30). Since these retinal waves cause dopamine release (5) we might expect light to increase vitreal dopamine concentration. However, a one-hour light pulse may not have been long enough to increase overall dopamine concentration *via* an increase in wave duration. Future work will assess the effects of longer light exposure on vitreal dopamine levels.

The emergence of light induced DOPAC release at P16 correlates with the increase in complexity of the dopaminergic cell dendritic/axonal plexus. We have previously suggested that light-inputs to dopaminergic cells occur locally at varicosities on TH cell processes (7). This hypothesis could support the data that we show here that while dopamine release in development is widespread, light-induced dopamine release may specifically rely on synapses formed on the dense plexus of processes that develop from P16-P30 (**Figure 3C**). The development of the TH cell plexus has also been shown in other mammalian species with this change in complexity particularly noted following eye-opening (8, 21, 22, 31). Together, these data suggest that dopamine is an important neuromodulator present in the retina prior to eye-opening, but its release in response to light is a feature that subserves image-forming vision specifically, given that it does not occur prior to eye-opening.

Finally, we show that light-activation of the retina is widespread throughout all ages prior to eye-opening and is driven exclusively by melanopsin. While light activation of Opn5, expressed in ganglion cells, has been described at these postnatal ages (29), we show here that this light activation does not cause c-fos activation. We were surprised to see the pattern of c-fos expression at P3 restricted to the dorsal retina despite melanopsin expression in RGCs in ventral retina. The ventral ipRGCs may still be activated by light, but our data suggests the strength of this activation, or at least the calcium response, is greater in the dorsal retina. Light responses of ipRGCs have been measured ~P3 but confinement of these response to the dorsal retina was not noted (32–34). Animals at P3 will receive a diffuse light stimulus at this age due to their closed eyes, meaning it is unlikely that this dorsal activation reflects the animal’s experience of the world. It is more likely that ipRGCs in the dorsal retina display phenotypic differences at this age to direct the development of retinal, or higher order visual circuits.

## Conclusion

Light-induced activation of dopaminergic amacrine cells, and dopamine release, in the mouse retina is tightly linked to eye-opening and the maturation of classical photoreceptor activation. While we show that melanopsin containing ipRGCs are strongly activated by light prior to eye-opening, they do not cause c-fos activation of dopaminergic cells or light-induced dopamine/DOPAC release. However, dopamine concentration of the vitreous is high in early development, under both dark and light conditions, indicating a significant role for this neuromodulator in retina/eye development. However, the role for dopamine in the retina likely shifts, following eye-opening, to driving light-adaptation of rod and cone circuits once they are mature.

## Data availability statement

The raw data supporting the conclusions of this article will be made available by the authors, without undue reservation.

## Ethics statement

The animal study was reviewed and approved by Western Sydney University Animal Care and Ethics Committee.

## Author contributions

VJ and MC wrote the manuscript and compiled the figures. MC designed the experiments and completed all the *in vivo* tissue removal. VJ, with help from SR, completed the immunohistochemistry and counting. PL in consultation with MM optimised and completed the LC-MS experiments. All authors read and contributed to the manuscript. All authors contributed to the article and approved the submitted version.
