# Supplementary material for: Light activation of the dopaminergic system occurs after eye-opening in the mouse retina
Source: Front Ophthalmol (Lausanne). 2023 May 9;3:1184627. doi: 10.3389/fopht.2023.1184627 (PMC11182289; doi:10.3389/fopht.2023.1184627)
Supplement: Supplementary file 1 [file DataSheet_1.pdf]

Supplementary Material

Light activation of the dopaminergic system occurs after eye-opening in the mouse retina

Vrinda Jain[1], Phillip Liang[1], Sushmitha Raja[1], Meena Mikhael[2], Morven A Cameron[1*]

*** Correspondence:**

Morven Cameron
m.cameron@westernsydney.edu.au

1.1 Supplementary Data 1

This excel sheet reports the spectrum of the white light used for light-pulsing. Included is the irradiance of this light at each wavelength (in $\mu W/cm^2$) and the calculation for conversion to photopic and scotopic candelas/m^2.

1.2 Supplementary Figure 1

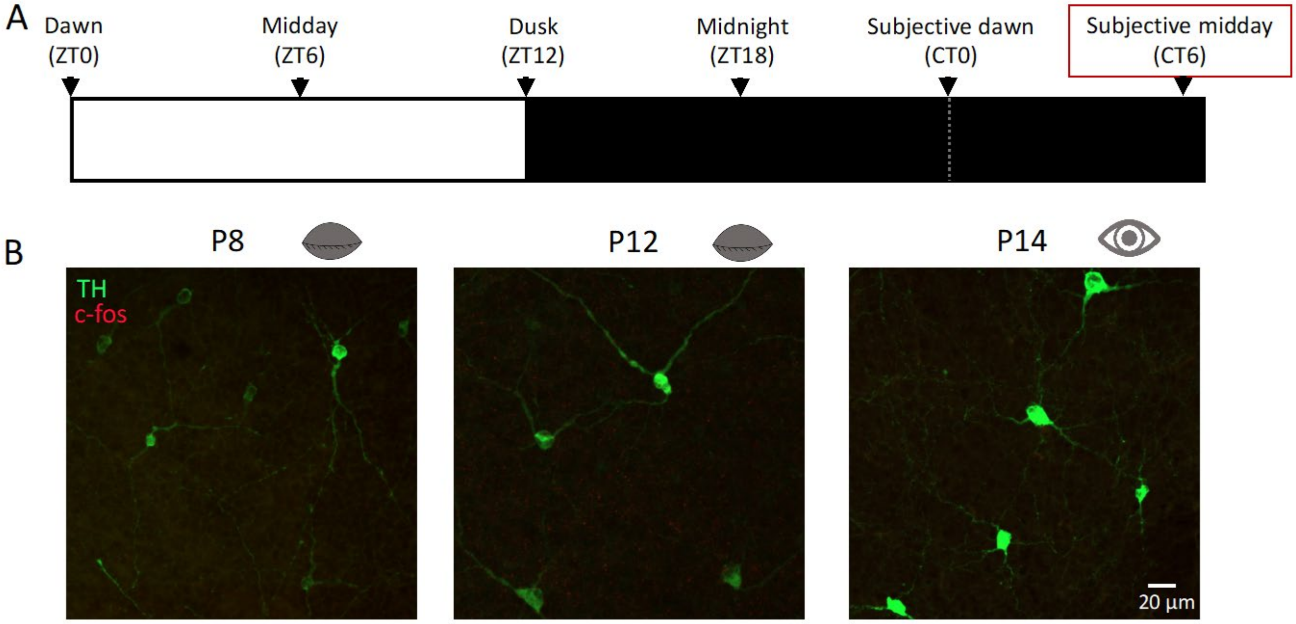

Supplementary Figure 1. *No c-fos expression is observed in the retina in the dark.* ***A****, Schematic representation of the light environment of the animals prior to tissue removal. Animals were dark-adapted from dusk the preceding day and then kept in the dark until subjective midday the following day (red box; CT6 ± 1.5 hrs) when ocular tissues were removed at under infrared illumination.* ***B****, Representative images of wholemount dark-adapted retinae stained for TH (green; dopaminergic*

cells) and c-fos (red). No c-fos expression was observed in dark-adapted retinae at postnatal day 8 (P8), P12 or P14.
